# When the Wounded Arrive

**Published:** 1917-06-30

**Authors:** Ward Muir

**Affiliations:** R.A.M.C. (T.) 3rd London General Hospital


### WHEN THE WOUNDED ARRIVE.

By LANCE-CORPORAL WARD MUIR, R.A.M.C. (T.), 3rd LONDON GENERAL HOSPITAL.

The receiving-hall of the hospital is its clearing-house for patients. It is a huge room, with a lofty and echoing roof, a little in the style of a church. Before the war, when the building was a school, this grandiose apartment no doubt witnessed speechifyings and prize-distributions. May the time be not far distant when it will once again be used for those observances! Meanwhile its vast floor is occupied by ranks of beds.

Those beds are generally untenanted. Visitors who, like the lady in the play, have taken the wrong turning, are apt to find themselves in the receiving-hall, and, gazing at its array of vacant beds, have been known to conclude that the hospital was empty. (As if any war hospital, in these times, could be empty!) But our patients have only a short acquaintanceship with the receiving-hall beds; these beds are momentary resting-places on their journey healthwards: they are not meant to lie in but to lie *on*. The three-score wards for which the receiving-hall is the clearing-house are the real destination of the patients; down long corridors, in wards far cosier because less ornate than this, the patient will find "his" bed ready for him, the bed which he is not to lie on but *in*.

We orderlies meet each convoy at the front door of the hospital. The walking-cases are the first to arrive—men who are either not ill enough, or not badly enough wounded, to need to be put on stretchers in ambulances. They come from the station in motor-cars supplied by that indefatigable body, the London Ambulance Column. The walking-case alights from his car, is conducted into the receiving-hall, and ten minutes later is in the bathroom. For the ritual of the bath must on no account be omitted—although now not so obviously imperative as in the early period of the war. Few patients reach us who have not first sojourned, either for a day or two or for weeks, in hospitals in France. They are therefore merely travel-stained, as you or I might be travel-stained after coming over from Dublin to Euston. The bath is thus a pleasure more than a necessity. Whereas there *was* an era when our guests came straight from only too populous trenches. . . .

"O.C. Baths," as the bathroom orderly was nicknamed, had to be circumspect in the performance of his job.

The few minutes which the walking-case spends in the receiving-hall are occupied (1) in drinking a cup of cocoa, and (2) in "having his particulars taken."

Poor soul!—he is weary of giving his "particulars." He has had to give them half-a-dozen times at least, perhaps more, since he left the Front. At the field dressing-station they wanted his particulars, at the clearing-station, on the train, at the base hospital, on another train, on the steamer, on the next train, and now in this English hospital. As he sits and comforts himself with cocoa, a "V.A.D." hovers at his elbow, intent on a printed sheet, the details of which she is rapidly filling-in with a pencil. For this is a card-index war, a colossal business of files and classifications, and ledgers and statistics, and registrations, an undertaking on a scale beside which Harrod's and Whiteley's, and Selfridge's and Wanamaker's, and the Magazin du Louvre, all rolled into one, would be a fleabite of simplicity. Ere the morrow shall have dawned our patient's military biography will be recounted, by various clerks, in I don't know how many different entries. If you are curious, refer to one of our volumes of the "Admission and Discharge Book: Field Service Army Book 27a." Open it at any of its closely written pages and see the host of ruled columns which the orderly in charge of it must inscroll with reference to each of the many thousands of patients who pass through our hospital per annum. The columns ask for his Regiment; Squadron, Battery, or Company; Number; Rank; Surname; Christian Name; Age; Length of Service; Completed Months with Field Force; Diseases (Wounds and Injuries are expressed by a number indicating their nature and whereabouts); Date of Admission; Date of Discharge or Transfer; Number of Days under Treatment; Number of Ward; Religion; and "Observations"—a space usually occupied by the name of the hospital ship upon which our friend crossed the Channel and the name of the convalescent home to which he went on bidding us adieu.

Having furnished the preliminary statements which lay the foundation of this compendious memoir, the walking-case thankfully finishes his cocoa, picks up the package of "blues" which has been put at his side, and departs, with his fellows, to the bathroom. Here he is tackled by the Pack Store orderlies, who take from him, and enter in their books, his khaki clothes. These he must leave in exchange for the blue slop uniform which, *pro tem.*, is to be his only wear. When he emerges from the bathroom he is attired in what is now England's most honourable livery—the royal blue of the war-hospital patient. And (though perhaps the matter is not mentioned to him in so many words) his own suit is already ticketed with an identification label and on its way to the fumigator. This is no reflection on the owner of the suit . . . but there are some things we don't talk about. Mr. Fumigator-Wallah is not the least busy of the more retiring members of a war-hospital staff. He is not in the limelight; but you might come to be very sad and sorry if he took it into his head to neglect his unapplauded part off-stage.

The walking-cases are still splashing and dressing in the bathroom, when the ambulances with the cot-cases begin to appear. Now is the orderlies' busy time. Each stretcher must be quickly but gently removed from the ambulance and carried into the receiving-hall.

Four orderlies haul the stretcher from its shelf in the ambulance; two orderlies then take its handles and carry it indoors. At the entrance to the receiving-hall they halt. The medical officer bends over the patient, glances at the label which is attached to him, and assigns him to a ward. (Certain types of cases go to certain groups of wards.) The attendant sergeant promptly picks up a metal ticket from a rack and lays it on the stretcher. The ticket has, punched on it, the number of the patient's ward and the number of the patient's bed in that ward. This ceremony completed, the orderlies proceed, with their burden, up the aisle between the beds in the receiving-hall.

Arrived at the bed, they lower their stretcher until it is at such a level that the patient, if he is active enough, can move off it on to the bed; if he is too weak to help himself he is lifted on to the bed by orderlies under the direction of the receiving-hall Sister. The stretcher is promptly removed and restored to its ambulance If the patient is in an exceptionally suffering condition he is not placed on the receiving-hall bed; instead—the medical officer having given his permission—his stretcher is put on a wheeled trolley and he is taken straight away to his ward, so that he will only undergo one shift of position between the ambulance and his destination. The majority of stretcher-cases, however, reach us in a by no means desperate state, for, as I say, they seldom come to England without having been treated previously at a base abroad (except during the periods of heavy fighting). And it is remarkable how often the patient refuses help in getting off the stretcher on to the bed. He may be a cocoon of bandages, but he will courageously heave himself overboard, from stretcher to bed, with a gay *wallop* which would be deemed rash even in a person in perfect health. Our receiving-hall, at a big intake of wounded, when every bed bears its poor victim of the war, presents a spectacle which might give the philosopher food for thought; but I suspect that, if he regarded its actualities rather than his own preconceptions, what would impress him more than the sadness would be on the one hand the kindliness, brisk but not officious, of the staff, and, on the other, the spontaneous geniality of the occupants of the beds. The orderlies can spare little time for talk, but the few chats that they are able to have with patients whom they are helping to change their clothes, or to whom they are proffering the inevitable cocoa (which is a cocktail, as it were, prior to the meal which will be served in the men's own ward), are punctuated by jokes and laughter rather than the long-visaged "sympathy" which the outsider might—quite wrongly—have pictured as appropriate to such an assemblage.

The stretcher-case, before he is taken to his ward, must also "give his particulars," must also be interviewed by the Pack Store officials, and must also have assigned to him his blue uniform (wherewith are a shirt, a cravat, slippers, and socks) in anticipation of the time when he shall be able to use his feet again and promenade our corridors and grounds. He receives the customary packet of cigarettes (probably the second, for he often gets one at the railway station too), and then, on another stretcher, mounted on a trolley, is wheeled off to his ward. Here, bestowed in bed at last, we leave him to his blanket-bath, his meal, his temperature-taking and chart filling-in by the Sister, his visit from the doctor, and all the rest of it. For the moment we see no more of him: we must race back to the receiving-hall, and, if there are no more patients to take away, return the trolley to its proper nook, put straight the blankets and pillows on the beds, sweep the floor, and tidy up generally, in readiness for the next convoy's advent.

Presently the huge room, beneath its dim arched ceiling, is silent and empty once more. The four ranks of beds, without a crease on their brown blankets, are bare of occupants. The Sister and her probationers have vanished. The Pack Store orderlies have carried off their loot of dirty khaki tunics and trousers for the fumigator. The clerical V.A.D.s have gone to enter "particulars" in ledgers and card-indices. The cookhouse people have removed their cocoa urn. The sergeant is inspecting the metal ward-tickets left in his rack. A glance at them tells him how many beds, and which beds, are free in the hospital; for the tickets have no duplicates; any given ticket can only reappear in the rack when the bed which it connotes is out of use and awaiting a newcomer; the ticket hangs from a nail in the wall beside the patient's bed just so long as that bed is tenanted. So the rack of metal tickets might almost take the place of that important document, of which a freshly-compiled edition is typed every morning, the Empty Bed List; and the sergeant is meditative as he sorts into the rack the tickets which have newly been sent in from the Sisters of wards where there have been departures. "Not much room in the eye-wound wards," he ponders; or, "A lot of empties in the medicals." And then . . . the tinkle of the telephone. . .

"Another convoy expected at 6.15! Twenty walking-cases and seventeen cots. Right you are!"

And at 6.15 the party of orderlies will be back again at the front door, again the motor-cars will stream up the drive, again the ambulances will come with their stretchers, and again the receiving-hall will awaken from its interlude of silence to echo with the activities incidental to a clearing-house of those damaged human bundles which are the *raison d'être* of our great war hospital.

